# Chronic noncommunicable diseases and population surveys

**DOI:** 10.1590/S1518-8787.201705100supl1ed

**Published:** 2017-06-01

**Authors:** Euclides Ayres de Castilho, Moisés Goldbaum

**Affiliations:** I Departamento de Medicina Preventiva. Faculdade de Medicina. Universidade de São Paulo. São Paulo, SP, Brasil

The epidemiological method, with increasing importance in the area of general health and, in particular, of Public Health, is a relevant field of study. It subsidizes, among other aspects, the definition of public policies on health. Among its several study designs, cross-sectional (or prevalence) studies are robust tools to describe living conditions and health situations of populations and specific groups.

Of these studies, health surveys stand out for having various modalities and widespread use in several countries, whether of household base aimed at population groups or morbidities. In Brazil, we highlight the institutionalization of national or regional surveys, whose regularity has offered important information for knowing the health status of our population and thus providing substantial elements to follow the evolutionary trends and subsidize the managers in their decision-making process.

The surveys conducted by the Brazilian Institute of Geography and Statistics (IBGE), such as the National Household Sample Survey (PNAD), the Household Budget Survey (POF), and the National Survey of School Health (PeNSE), are highlighted examples of information databases that allow consistently describing the situations experienced by our population. At their side, we must register the annual surveys conducted via landline (VIGITEL), by the partnership between Brazilian Ministry of Health and Universidade de São Paulo.

More recently, in the partnership between the Brazilian Ministry of Health and IBGE – which had already conducted PeNSE –, the unprecedented and first National Survey on Health (PNS) was performed, aiming to obtain the perception of health conditions, lifestyles, and chronic diseases in Brazil, in the macroregions and Federative Units. The data, presented to everyone in the IBGE website, have been stimulating and challenging our researchers to promote analyses aimed at publicizing and disseminating the information on the survey objects. With this supplement, *Revista de Saúde Pública* (RSP) opens the opportunity for broad dissemination of articles, whose analysis objects comprise the data registered on the PNS database.

The consultation to this supplement will allow readers to become aware of different aspects related to the Brazilian population health: chronic diseases, with emphasis on hypertension and diabetes, and their involvement in the use of health services; older adults, studied from the perspective of use of health services and of the impact represented by informal care; cancer, addressed by the analysis of breast cancer; inequalities in the life expectancy of Brazilians; analysis of eating habits in children; studies on depression and also musculoskeletal diseases. This timely supplement opened an equally important space to the dissemination of aspects of improvement of VIGITEL.

These situations indicate the variety of possibilities of analysis and production of knowledge about population health that PNS provides. RSP, in presenting this supplement, expresses its satisfaction by fulfilling its goals of disclosure and dissemination of scientific knowledge. It expects this material will guide readers and managers in conducting their social and health responsibilities and encourage the community of researchers and managers to address the PNS database and explore it thoroughly. With this, we expect to support the production of many other analyses compromised in covering gaps of knowledge and providing materials to reduce the immense inequalities and inequities experienced by the Brazilian society.

